# Behavioral adaptations of *Caenorhabditis elegans* against pathogenic threats

**DOI:** 10.7717/peerj.19294

**Published:** 2025-04-14

**Authors:** Xin Zhao, Xinyu Li, Jiayi Gao, Shi Shen, Wei Zou

**Affiliations:** 1 Kunming Medical University, School of Public Health, Kunming, Yunnan, China; 2 Xi’an Public Health Center, Infection Control Office, Xi’an, Shaanxi, China

**Keywords:** *Caenorhabditis elegans*, Pathogenic bacteria, Behavioral plasticity mechanisms

## Abstract

This review examines the behavioral adaptation mechanisms of *Caenorhabditis elegans* in response to pathogenic bacterial threats, emphasizing their ecological significance. It systematically explores how mechanisms such as avoidance behavior, transgenerational learning, and forgetting enable *C. elegans* to optimize its survival and reproductive strategies within dynamic microbial environments. *C. elegans* detects harmful signals through chemosensation and initiates avoidance behaviors. Simultaneously, it manages environmental adaptation and energy allocation through transgenerational memory and forgetting, allowing *C. elegans* to cope with selective pressures from environmental fluctuations. In contrast, pathogenic bacteria such as *Pseudomonas aeruginosa* and *Salmonella* influence *C. elegans* behavior through strategies such as toxin release and biofilm formation, highlighting the complex co-evolutionary dynamics between hosts and pathogens. Additionally, these pathogens employ “Trojan Horse-like” and “Worm Star” mechanisms to kill *C. elegans*, further complicating host-pathogen interactions. These processes are driven by behavioral adaptations, biochemical signaling, and evolutionary pressures, which emphasize the ecological niche of *C. elegans* within microbial ecosystems. *C. elegans* serves as a valuable model for studying host-pathogen interactions. This study provides crucial theoretical insights into adaptive evolution and ecosystem dynamics, offering valuable guidance for the development of biocontrol strategies and the effective management of microbial ecosystems.

## Introduction

*Caenorhabditis elegans* (commonly abbreviated as *C. elegans*) serves as an essential model for studying behavioral adaptation and ecological interactions, demonstrating remarkable capabilities in sensing, learning, and adapting to complex microbial environments. Its transparent body and fully sequenced genome provide unique advantages for real-time imaging and in-depth exploration of gene functions (Leung et al., 2008; Wernike, van Oostende & Piekny, 2014). These characteristics position *C. elegans* as a leading model organism in the study of cellular differentiation, gene function, and environmental adaptability. For example, research on the behavioral adaptations of *C. elegans* has elucidated its use of avoidance behavior, memory-based learning, and forgetting mechanisms in response to toxic metabolites and pathogenic bacteria, thereby augmenting its survival capacity (Meisel & Kim, 2014; Vogt & Hobert, 2017; Zhang, Lu & Bargmann, 2005).

In nature, *C. elegans* primarily inhabits decaying plant material, compost heaps, and rotting fruits by feeding on bacteria and fungi (Andersen et al., 2012; Crombie et al., 2019). These provide the range of nutrients needed for growth and reproduction and also form the basis of its behavioral adaptations to life in microbially dense environments (Barrière & Félix, 2005). *C. elegans* usually dwells in a state of partial dormancy in natural environments. When its larvae encounter an excess supply of bacterial foods, for example, they develop swiftly into sexually mature adults with high reproduction rates that rapidly consume the available bacterial biomass in large proportions (Bongers, 1990). Adaptive behaviors displayed by *C. elegans* include the capacity to differentiate between beneficial and harmful microorganisms. Through feeding preferences and pathogen avoidance, a balance between survival and reproduction is maintained in the dynamic microbial communities in which it resides (Joshua et al., 2003; Köthe et al., 2003; Samuel et al., 2016).

Pathogenic bacteria, as integral components of ecosystems, exert a profound impact on *C. elegans* behavior through mechanisms such as biofilm formation, quorum sensing, and the release of virulence factors. *Yersinia pseudotuberculosis* alters its infection dynamics *via* biofilm formation, whereas *Pseudomonas aeruginosa* adjusts its virulence through quorum sensing signaling (Schulenburg & Ewbank, 2007; Tran et al., 2017). In response, the complicated host-pathogen interactions in *e.g*., *C. elegans* have been represented through changes in behavior, the ability to sense pathogenic signals, and deploying survival strategies such as physiological resistance to pathogens (Irazoqui et al., 2010). This review is supposed to explore, analyze, and investigate how *C. elegans* adapts to bacterial pathogenic threats through behaviors such as avoidance, learning, and forgetting, and how such behaviors impact the ecological interactions and co-evolutionary dynamics between *C. elegans* and pathogenic bacteria.

## Audience

The audience for this review includes researchers and scientists studying behavioral adaptation, host-pathogen interactions, and ecological dynamics, particularly those focused on *C. elegans* as a model organism.

## Survey methodology

We conducted a search in the PubMed databases for articles published before January 2, 2025, focusing on the relationship between behavioral adaptation and the nervous system in *C. elegans* in response to pathogenic infections. The search was performed using the following keywords: ((*Caenorhabditis elegans*) AND (pathogen)) AND (memory) AND (“forgetting behavior” OR “Trojan Horse-like” OR “Worm Star” OR “killing”). To identify additional relevant publications, we also examined the references cited in the articles retrieved. Studies were included based on the following criteria: research discussing the mechanisms by which pathogenic bacteria kill *C. elegans* and the associated “Trojan Horse-like” or “Worm Star” mechanisms, and studies investigating *C. elegans* cross-generational learning, avoidance memory, forgetting, exploration behavior, maternal mortality, and egg-laying response behavior, along with the genes, signaling molecules, or neurons involved in these behaviors.

## Behavioral adaptation of *c. elegans*: mechanisms for managing pathogenic bacteria

Adaptive responses in *C. elegans* include sensory management, neuropeptides, behavioral adaptability, immune processes, and microbiome interactions that all give this animal a remarkable survival advantage in challenging conditions (Harel, Nasser & Stern, 2024; Kumar et al., 2020). These approaches represent an important strategy for investigating host-pathogen interactions and the development of immune responses because they can show the ability of an organism to adapt to complex environmental stressors. Behavioral plasticity of *C. elegans* is manifested in its flexible responses to multiple sensory stimuli, involving odors, salts, mechanical stimulus, and even temperature change (Watteyne et al., 2024; Zhang, Iino & Schafer, 2024). Such sensory plasticity allows the worm *C. elegans* to alter its behaviour in reaction to experiential inputs, thus leading to its efficient adaptation towards environmental threats. Interestingly, within its complex sensory responses fall mechanisms of O^2^-sensing (McGrath et al., 2009; Valperga & de Bono, 2022) and temperature learning (Yoon et al., 2017) (Table 1). The thermotactic behavior of *C. elegans* exemplifies its ability to remember optimal temperatures and migrate toward these favorable thermal zones. Differences in the thermotactic strains CB4854 and CB4857 reveal that *C. elegans* can optimize behavioral patterns according to environmental conditions (Anderson et al., 2011; Félix & Duveau, 2012). While on a bacterial lawn, equivalent to a foraging patch, *C. elegans* has three main behavioral states: roaming, dwelling, and quiescence (Ben Arous, Laffont & Chatenay, 2009; Fujiwara, Sengupta & McIntire, 2002; Hill et al., 2014). Its extended states of roaming represent an important foraging behavior, regulated by the neuropeptide PDF-1 and its receptor PDFR-1 (Flavell et al., 2013).

**Table 1:** Mechanisms and behavioral outcomes of *C. elegans* response to pathogenic bacteria.

Number	Pathogenic bacteria	Mechanism	Behaviour	Cite
1	PA14	Pathogen exposure in *C. elegans* induces chemoreceptor STR-44 in AWA sensory neurons, altering pheromone responses to suppress avoidance and promote mating, enhancing genetic diversity and adaptation.	Avoidance suppression and mating promotion	Lee et al. (2022)
2	PA14	Locomotion enhances aversive olfactory learning by activating mechanoreceptors in motor neurons, which transmit proprioceptive information to interneurons through gap junctions.	Locomotor activity-induced learning	Zhan et al. (2023)
3	PA14	Expression of the TGF-β ligand DAF-7 in ASI sensory neurons, along with Piwi Argonaute homolog PRG-1 and its downstream components, is required for transgenerational inheritance of avoidance behavior and ASI daf-7 expression.	Avoidance behavior, Genetic adaptation	Moore, Kaletsky & Murphy (2019)
4	PA14	Exposure to PA14 during the larval stage induces a lasting aversion memory *via* regulation of tyramine and specific neurons (such as RIA), dependent on the SER-2 receptor.	Long-term avoidance memory	Jin, Pokala & Bargmann (2016)
5	PA14	Disruption of core cellular activities (translation, respiration, and protein turnover) triggers behavioral avoidance of normally attractive bacteria through a neuroendocrine axis involving detoxification, immune responses, and signaling pathways.	Avoidance behavior	Melo & Ruvkun (2012)
6	PA14	Aversive olfactory learning requires AWB and AWC olfactory sensory neurons.	Avoidance behavior	Ha et al. (2010)
7	PA14	CYSL-1 and CYSL-2, cysteine dehydrogenases, mediate parental exposure to pathogenic bacteria to enhance offspring immunity.	Avoidance behavior, Genetic adaptation	Burton et al. (2020)
8	PA14; Oxide dismutase -1 (SOD-1)	*C. elegans* utilizes the ROS-sensing enzyme SOD-1 in gustatory neuron ASER to regulate aversive behavior, enabling an adaptive delayed response to pathogens.	Avoidance behavior	Horspool & Chang (2017)
9	Bacterial toxic metabolites tambjamine and violacein	*C. elegans* avoids toxic sulforaphane YP1 through innate aversion. Violacein’s learned avoidance is specific and reversible, mediated by the olfactory system and decreases when serotonin is lacking.	Avoidance behavior	Ballestriero et al. (2016)
10	Streptomyces	*C. elegans* detects and avoids Streptomyces producing toxin using chemosensory receptor SRB-6.	Avoidance behavior	Tran et al. (2017)
11	*Serratia marcescens*	TLR signal transduction affects *C. elegans*’ behavioral response to *Serratia marcescens*.	Avoidance behavior	Brandt & Ringstad (2015)
12	*Vibrio cholerae*	*Vibrio cholerae* produces a quorum sensing signal molecule CAI-1, detected by *C. elegans via* AWCON chemosensory neurons.	Exploration behavior	Werner et al. (2014)
13	Secondary metabolites of *Pseudomonas aeruginosa*	*C. elegans* detects secondary metabolites of *Pseudomonas aeruginosa* through chemical sensing to regulate neuroendocrine signals and promote avoidance behavior.	Avoidance behavior	Meisel et al. (2014)
14	*Pseudomonas aeruginosa* and *Salmonella enterica* serotype Typhimurium MST1	Under conditions of hunger, rising temperature, or crowding, *C. elegans* enters diapause and becomes dauer larvae.	Diapause entry	Palominos et al. (2017)
15	Bacterial metabolite viologen	*C. elegans* shows behavioral adaptability, such as matrix biting, to cope with the toxic effect of bacterial metabolite violacein.	Maternal mortality and egg-laying response	Yoon et al. (2020)

Behavioral adaptability constitutes a significant characteristic of *C. elegans*. In response to pathogenic bacterial stress, *C. elegans* demonstrates a range of adaptive strategies, such as avoidance behavior, alterations in foraging preferences, maternally-induced inhibition of egg-laying, delayed developmental processes, and transgenerational learning (Table 1). Empirical studies have indicated that following exposure to pathogenic bacteria, the acquired avoidance behavior in adult *C. elegans* can be transmitted to subsequent generations *via* RNA interference (RNAi), exemplifying a mechanism of transgenerational adaptation (Vidal-Gadea et al., 2011). *C. elegans* effectively responds to different environments by flexibly switching between crawling and swimming behaviors, further highlighting its ecological adaptability (Vidal-Gadea et al., 2011).

### *C. elegans* adapts to pathogenic bacterial threats *via* associative learning and memory in avoidance behavior

Avoidance behavior constitutes a crucial adaptive strategy for *C. elegans* in response to pathogenic bacteria, predominantly encompassing learned avoidance and modifications in olfactory preferences to minimize pathogen contact. Upon exposure to *Pseudomonas aeruginosa* PA14, *C. elegans* exhibits a biphasic avoidance response: an initial attraction phase succeeded by a repulsion phase. Initially, *C. elegans* displays a natural attraction to PA14; however, after 4–6 h of exposure, it initiates avoidance of PA14 through an acquired avoidance mechanism (Zhang, Lu & Bargmann, 2005). This process entails a minimum of three neural circuits: the AWB-AWC sensory-motor circuit facilitates initial attraction and subsequent avoidance responses (Ha et al., 2010; Lei et al., 2024), whereas the reflexive aversion circuit, mediated by AWB neurons, and the ADF regulatory circuit predominantly regulate the learned avoidance response (Doroquez et al., 2014). Furthermore, *C. elegans* demonstrates alterations in behavior following exposure to pathogens like *Serratia marcescens* and *Pseudomonas aeruginosa*; with extended exposure, the tendency transitions from attraction to avoidance (Filipowicz, Lalsiamthara & Aballay, 2022; Meisel & Kim, 2014). *C. elegans* may modify its olfactory preferences according to previous experiences, so evading possible diseases, which is essential for its survival (Petersen et al., 2021; Sengupta et al., 2024). Following exposure to certain stimuli, *C. elegans* exhibits olfactory imprinting, whereby early-life exposure to PA14 results in enduring behavioral modifications (Vogt & Hobert, 2017). This process is governed by brain systems, including the function of CREB in facilitating long-term responses (Timbers & Rankin, 2011). Research indicates that certain neuropeptide receptors, notably NPR-1, are essential in modulating these behaviors, which are influenced by the interactions between *C. elegans* and different types of bacteria (Reddy et al., 2011).

### Forgetting and transgenerational inheritance in *C. elegans*

Besides avoidance behavior driven by learning and memory imprinting, *C. elegans* has adaptive ability over various time scales when confronted with dangerous germs. Forgetting constitutes a fundamental behavioral response of *C. elegans* when exposed to pathogenic bacteria. As shown in Table 1, *C. elegans* initially shows a preference after being exposed to PA14 (Zhang, Lu & Bargmann, 2005); however, this preference transitions to avoidance. Notably, this avoidance behavior is transient, as it dissipates after one hour, resulting in *C. elegans* once again being attracted to PA14 (Hadziselimovic et al., 2014; Liu et al., 2022). *C. elegans* can modulate the forgetting process *via* the minor G-protein RAC-2 and JNK-1 signaling pathways (Bai et al., 2022). Furthermore, driven by salt ions, actin and the RNA-binding protein Musashi are pivotal in the forgetting process, suggesting that forgetting is an active, signal-regulated phenomenon (Hadziselimovic et al., 2014; Kitazono et al., 2017). The forgetting behavior of *C. elegans* is not simply a reduction in memory, but an active process governed by several signaling routes, chemical processes, and intricate neuronal connections (Kitazono et al., 2017).

In addition to forgetting, *C. elegans* demonstrates the ability to respond to environmental stressors through transgenerational inheritance mechanisms. Research has shown that parental exposure to pathogens can lead to heritable changes in offspring traits, improving their resistance to subsequent infections. For instance, exposure to the pathogen *Pseudomonas vranovensis* enhances offspring immunity through a mechanism dependent on the genes CYSL-1, CYSL-2, and RHY-1 (Burton et al., 2020). Vitamin B12, an essential nutrient for *C. elegans* growth and development, also plays a key role in transgenerational effects. Parental exposure to vitamin B12 or vitamin B12-producing bacteria accelerates offspring growth and enhances their tolerance to infections, with these effects relying on the methionine biosynthesis and propionyl-CoA breakdown pathways (Willis et al., 2024; Zečić, Dhondt & Braeckman, 2019). Moreover, various experiences in the parental generation, such as dietary restriction, osmotic stress, temperature changes, olfactory imprinting, and prolonged starvation, can profoundly affect the physiology of their offspring. Some of these effects persist for multiple generations and are mediated through small RNA regulation (Liu & Zhang, 2020). Additionally, adult worms can pass on learned pathogen-avoidance behaviors to their progeny. This transmission involves the RNA interference (RNAi) pathway, the piRNA pathway, and the coordinated action of ASI neurons and the reproductive system (Kaletsky et al., 2020). For example, exposure to small RNAs (sRNAs) from *Pseudomonas aeruginosa* PA14 induces pathogen-avoidance behaviors in *C. elegans*, which are inherited for up to four generations (Kaletsky et al., 2020). Similarly, a pathogenic *Pseudomonas vranovensis* strain from the *C. elegans* microbiota induces learned avoidance behavior in worms, which is inherited through bacterial small RNAs for four generations, supporting the idea that such transgenerational behavioral effects also occur in the wild (Sengupta et al., 2024). These findings suggest that the adaptive responses of *C. elegans* go beyond individual behavioral adjustments, influencing offspring survival through complex genetic and molecular mechanisms, thereby enhancing their ability to cope with pathogenic threats.

### Activation of physiological cell defenses in *C. elegans* to combat pathogenic bacterial attacks

Forgetting and transgenerational inheritance exemplify the genetic and temporal continuity of behavioral adaptations in *C. elegans*. However, within the survival strategies of *C. elegans*, behavioral adaptations and physiological defenses are not discrete phenomena; instead, they are interconnected through complex signaling pathways and physiological mechanisms. In *C. elegans*, behavioral state or “context” is largely defined by food availability and is translated by both synaptic and extrasynaptic monoaminergic/peptidergic signaling to modulate the sensory-mediated locomotory decision-making associated with nociception (Komuniecki et al., 2014). Through intricate interactions between sensory receptors and signaling pathways, *C. elegans* possesses the capability to detect and evade harmful chemical stimuli, a function essential for its survival (Chaubey et al., 2023; Mills et al., 2016). This sensory response is influenced by previous experiences, enabling *C. elegans* to integrate current behavioral states with past experiences to refine its avoidance strategies under thermal stress (Byrne Rodgers & Ryu, 2020). The processing of information by sensory neurons further augments the ability of *C. elegans* to discern environmental signals. β-arrestin-mediated desensitization of olfactory receptor neurons helps *C. elegans* regulate its behavioral responses when exposed to various olfactory stimuli (Merritt et al., 2022; Zhao & Wang, 2012).

Beyond sensory regulation, *C. elegans* augments its pathogen resistance through the activation of its innate immune system. This immune defense is mediated by several signaling pathways, including the transforming growth factor-beta (TGF-β) pathway, the DAF-2/16 insulin-like signaling pathway, the p38 mitogen-activated protein kinase (MAPK) pathway, and the unfolded protein response (UPR) pathways (Adair & Douglas, 2017; Kwon, Kim & Lee, 2018; Radeke & Herman, 2021; Wong et al., 2007). Among these pathways, the p38 MAPK pathway is of particular importance, as mutations within this pathway result in a markedly increased susceptibility to pathogens in *C. elegans* (Chen et al., 2017; Chou et al., 2013; Osman et al., 2018). The study found that deletion of the fepB gene in *S. typhimurium* reduced its pathogenicity and triggered enhanced *C. elegans* dauer formation *via* the TGF-β pathway, while also improving worm survival and revealing the bacteria role as both a nutrient source and a signal regulating host physiology and host-pathogen interactions (Mallick et al., 2022). Furthermore, small RNAs, such as *let-7*, are crucial in modulating immune responses, thereby elucidating the intricate molecular network through which *C. elegans* adapts to pathogenic challenges (Zhi et al., 2017). *Pseudomonas aeruginosa* disrupts iron homeostasis in *Caenorhabditis elegans*, triggering a hypoxic response that ultimately results in the death of the organism (Kirienko et al., 2013).

In response to pathogenic threats present in the environment, *C. elegans* employs developmental regulatory strategies. Upon detecting pathogen-associated signals, *C. elegans* can enter a state of developmental arrest, a mechanism that enhances offspring survival in environments with prevalent pathogens (Palominos et al., 2017). This strategy is generally activated under conditions of food scarcity or high population density, wherein development is temporarily halted until environmental conditions improve, thereby conferring a survival advantage to both individuals and populations (Golden & Riddle, 1984). Moreover, the intestinal microbiota of *C. elegans* is integral to pathogen resistance and host immune function. The intestinal milieu of *C. elegans* acts as a selective filter, shaping core microbial communities from the diverse bacterial populations present in natural substrates (Berg et al., 2016). These microbial communities not only modulate host physiological processes but also bolster pathogen resistance through their metabolic activities. For instance, the non-pathogenic bacterium *Pseudomonas putida* enhances the resistance of *C. elegans* to *Pseudomonas aeruginosa* by secreting beneficial metabolites (Kissoyan et al., 2019). Conversely, certain pathogens, including *Pseudomonas aeruginosa* and *Enterococcus faecalis*, possess the ability to adapt to the host internal environment by neutralizing the intestinal pH, which facilitates a stable infection within the host (Benomar et al., 2020). These findings underscore the dual role of gut microbiota in host-pathogen interactions and underscore the significance of microbial diversity in influencing the ecological dynamics of *C. elegans* (Table 1).

Genetic diversity is important in ecological adaptation and survival. Significant genetic variation among the *C. elegans* populations exists across geographic regions, mostly driven by local adaptation to different environmental conditions (Braendle & Paaby, 2024; Crombie et al., 2022; Salas et al., 2022). For example, The genetic diversity of the *C. elegans* population in Hawaii is higher than in other regions, indicating that unique ecological pressures have contributed to genetic differentiation (Crombie et al., 2022). Host-transposon interactions further drive rapid genome diversification in natural populations, fostering evolutionary innovations in gene and splicing mechanisms (Zhang, Félix & Andersen, 2024). Moreover, in *C. elegans*, the nematode gene *rml-3*, acquired through interspecies horizontal gene transfer (iHGT) from bacteria, contributes to cuticle integrity and resistance to environmental stresses. This serves as an additional example of how iHGT has impacted metazoan evolution by incorporating bacterial genes that confer novel adaptive capabilities (Pandey et al., 2023). This genetic diversity is indicative of historical population dynamics and underscores the continuous influence of gene flow and selective pressures on niche adaptation (Lee et al., 2021). Moreover, research into the mechanisms of gene regulation in *C. elegans* thus underline how connected regulatory modules drive neuronal identity, development, and postembryonic diversification to provide a view on how neuronal diversity could have evolved (Poole, Flames & Cochella, 2024).

*C. elegans* exhibits a range of adaptive responses upon exposure to pathogenic bacterial attacks. These include behavioral avoidance of pathogens, active cellular defense mechanisms against microbial invasion, such as the expression of antimicrobial peptides or the mobilization of immune cells, and tolerance to pathogens (Schneider & Ayres, 2008; Schulenburg, Kurz & Ewbank, 2004). These collectively represent a multi-layered adaptive strategy. These adaptive responses, however, do not occur in a vacuum. Pathogenic bacteria actively manipulate the behavior and physiology of *C. elegans* by several ecological tactics, thus making it an ecological interaction.

### The ecological role of pathogenic bacteria in the behavioral adaptation of *C. elegans*

Pathogenic bacteria are integral to ecological dynamics through their interactions with *C. elegans*. The responses of *C. elegans* to diverse stressors, such as oxidative stress and toxin exposure, have been extensively investigated, yielding significant insights into mechanisms of detoxification and stress resistance (Stupp et al., 2013). These interactions are facilitated by the secretion of metabolites and toxins, as well as biofilm formation, which collectively influence the dynamics of microbial communities and drive the evolutionary adaptations of the host.

### Toxin secretion by pathogenic bacteria

**Figure 1:**
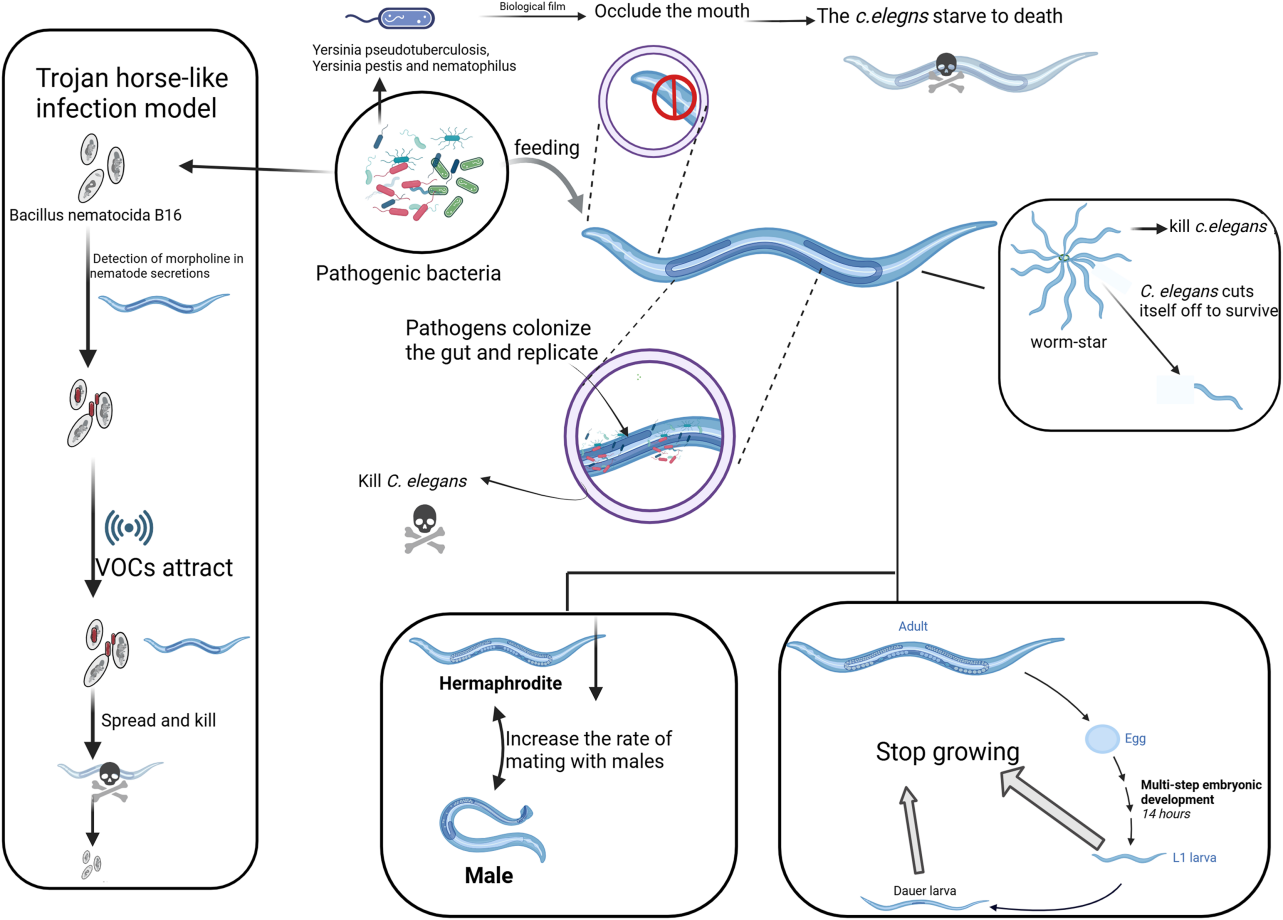
Different strategies of pathogenic bacteria attacking *C. elegans*. The main strategies employed by pathogenic bacteria to attack *C. elegans*, including toxin secretion, biofilm formation, and manipulation of behavior. On the left, the “Trojan Horse” mechanism shows how *Bacillus* bacteria attract *C. elegans* through its chemotactic response. The “worm star” phenomenon, depicted on the right, illustrates the mechanism by which pathogenic bacteria induce aggregation and mortality in *C. elegans* through the secretion of adhesive factors. The figure elucidates various strategies employed by pathogens to lethally interact with and propagate within *C. elegans*, as evidenced by the involvement of the nematode’s head, gut, reproductive system, and tail. Created with BioRender.com.

To evade predation, numerous bacterial species have developed a range of defensive mechanisms, such as the synthesis of deterrents or toxic metabolites. A predominant strategy utilized by pathogenic bacteria involves the secretion of toxins. Notably, species such as *Pseudomonas aeruginosa*, *Serratia marcescens*, *Bacillus thuringiensis*, *Bacillus cereus*, *Bacillus subtilis, Bacillus anthracis*, and *Bacillus megaterium*, among others, produce toxins that interfere with the physiological processes of *C. elegans* (Bird et al., 2015; Kaletsky et al., 2020; Niu et al., 2015; Rae et al., 2010; Zheng et al., 2016). Different bacterial genera use various mechanisms to efficiently and rapidly kill *C. elegans* (Khan, Jain & Oloketuyi, 2018). Pathogenic bacteria modulate the behavior of *C. elegans* not only through the release of toxins but also *via* the production of signaling molecules. For instance, bacteria that synthesize indole have been demonstrated to modify the feeding and reproductive behaviors of *C. elegans*, while indole-deficient strains exert toxic effects that diminish egg-laying (Lee et al., 2017). Additionally, the small RNA molecule P11 regulates ammonia production, which in turn affects the attraction of *C. elegans* to *Pseudomonas aeruginosa*. This indicates that nitrogen assimilation is pivotal in cross-boundary signaling and the interaction between bacteria and their host (Marogi et al., 2024). In natural populations of *C. elegans*, self-fertilization predominates as the principal reproductive strategy. Nevertheless, instances of outcrossing are exceedingly infrequent, occurring at an approximate rate of 1% (Barrière & Félix, 2005). Empirical studies have demonstrated that exposure to the pathogen *Pseudomonas aeruginosa* PA14 results in an increased frequency of copulation between hermaphroditic *C. elegans* and males. Subsequent investigations have elucidated that this phenomenon is contingent upon the *str-44* gene within the AWA neurons (Zhan et al., 2023). These findings suggest that bacterial metabolites can exert a substantial influence on the ecological interactions between *C. elegans* and its microbial milieu (Fig. 1).

### Biofilm formation by pathogenic bacteria

Biofilm formation is another key aspect of the ecology of pathogenic bacteria. Bacteria such as *Pseudomonas aeruginosa* can form biofilms, providing them with a protective environment that helps resist host immune responses and antibiotics, thereby enhancing their pathogenicity (Fila et al., 2018). The biofilm matrix can also influence *C. elegans* behavior, as *C. elegans* may be attracted to or repelled by chemical signals released by bacteria forming the biofilm, thereby affecting their foraging strategies and survival ability (Dirksen et al., 2016). *Yersinia pseudotuberculosis, Yersinia pestis*, and *Wolbachia* are capable of forming biofilms around the head of *C. elegans*, obstructing its mouth and preventing it from ingesting bacteria (Fig. 1), leading to starvation and death (Darby et al., 2002; Drace & Darby, 2008). Pathogenic bacteria like *Pseudomonas aeruginosa* and *Salmonella* have been shown to form biofilms that significantly affect *C. elegans* survival and health. *Pseudomonas aeruginosa* enhances its surface adhesion ability through the filamentous Pf4 bacteriophage, promoting biofilm formation. This process leads to increased virulence in the *C. elegans* model (Schwartzkopf et al., 2023). In chronic infection models, colonization of the *C. elegans* digestive tract by *P. aeruginosa* is a significant contributor to mortality (Fig. 1), underscoring the essential role of biofilm formation in its pathogenicity (Tse-Kang et al., 2024). *Salmonella* biofilms have been shown to modulate the innate immune response of *C. elegans*, facilitating persistent infections that are observable within the *C. elegans* intestine (Desai et al., 2019).

The ability of pathogenic bacteria to manipulate the *C. elegans* response further complicates the interactions between the *C. elegans* and the pathogen. *Yersinia pseudotuberculosis* regulates its virulence factors through quorum sensing, promoting biofilm formation on *C. elegans* (Atkinson et al., 2011). The biofilm matrix itself can also interfere with *C. elegans* movement and predation behavior, indicating that biofilms are not only protective structures for bacteria but also actively influence host behavior (Chan et al., 2021). In contrast, some bacterial species (*e.g*., *Bacillus* subtilis) form biofilms that extend the lifespan of *C. elegans* by mechanisms such as downregulation of the insulin-like signaling pathway (Donato et al., 2017). This suggests that biofilm interactions are not universally harmful; certain beneficial bacteria can enhance host resistance to pathogens. The presence of beneficial biofilms may also contribute to the overall health of the host by competitively excluding pathogenic strains (Donato et al., 2017). The investigation of biofilms extends beyond fundamental science and holds substantial clinical significance. The application of quorum sensing inhibitors has demonstrated efficacy in obstructing biofilm formation and diminishing the virulence of *Pseudomonas aeruginosa*, thereby safeguarding *C. elegans* and potentially human cells from infection (Atkinson et al., 2011). Comprehending the mechanisms through which pathogenic bacteria form biofilms and interact with hosts can guide the development of innovative therapeutic strategies.

### “Trojan Horse-like” and “Worm Star” mechanisms of pathogenic bacteria in killing *C. elegans*

The “Trojan Horse-like” mechanism of *C. elegans* denotes a very elaborative means of action developed by the pathogenic bacterium *Bacillus nematocida* B16, through which this nematode can invade and kill its host. In the first place, bacteria sense some secretion of *C. elegans*, like morrill, and spore formation is initiated. Along with spore formation, the bacteria start emitting some VOCs as signaling molecules for *C. elegans* (Fig. 1). After penetrating the intestine, the spores germinate and multiply, killing the host. During this process, *B. nematocida* continues to reproduce and decompose the body of the nematode (Niu et al., 2010; Zhang et al., 2020).

This strategy is appropriately termed “Trojan Horse-like,” since the bacteria in this case appear as harmless, deceiving *C. elegans* and therefore allowing for a pathogenic attack. This interaction, however, has been developed to be much more complex rather than just a simple infection of the nematode by the bacterium. *Bacillus* can manipulate *C. elegans* chemotactic behaviors to its advantage to promote bacterial survival and proliferation (Zhang et al., 2016). The VOCs emitted by *Bacillus* attract *C. elegans* in a manner that is hospitable to bacterial colonization and persistence within the host (Niu et al., 2010). The “Trojan Horse-like” mechanism illustrates a high degree of complexity in microbial pathogenic strategy: through chemical signaling, *Bacillus* lures *C. elegans* for efficient colonization and exploitation, culminating in host death.

The “Worm Star” phenomenon, observed in host-pathogen interactions, refers to a biological event where pathogenic bacteria, such as Leucobacter species, “capture” *C. elegans*. This phenomenon occurs when *C. elegans* individuals become trapped together as a result of bacterial infection (Fig. 1). It underscores the susceptibility of *C. elegans* to pathogenic attacks while serving as a valuable model for investigating the evolutionary adaptations of *C. elegans* to combat pathogenic threats (Hodgkin et al., 2013). Upon exposure to certain bacterial strains, including *Leucobacter celer*, *C. elegans* initiates a defensive response characterized by the formation of “worm star” aggregates (Fig. 1). This phenomenon predominantly manifests at the posterior end of the *C. elegans*, where individuals adhere to one another, creating a star-like configuration. Consequently, the entrapped *C. elegans* succumb to asphyxiation or bacterial degradation (Clark & Hodgkin, 2015; Hodgkin, Clark & Gravato-Nobre, 2014). The mechanism behind the “Worm Star” phenomenon involves bacterial factors that promote adhesion between *C. elegans*. Interestingly, *C. elegans* exhibits a negative chemotactic response, where they actively sever parts of their bodies to escape from the pathogen (Hodgkin, 2019). Adult *C. elegans* that successfully escape from the “worm star” aggregates often show signs of wound healing, suggesting that they possess remarkable tissue repair capabilities following such traumatic events (Hodgkin, 2019). This research not only enhances our understanding of *C. elegans* as a model organism but also reveals the broader ecological and evolutionary significance of host-pathogen interactions.

### Host-pathogen counteractions and coevolution in ecological environments

**Figure 2:**
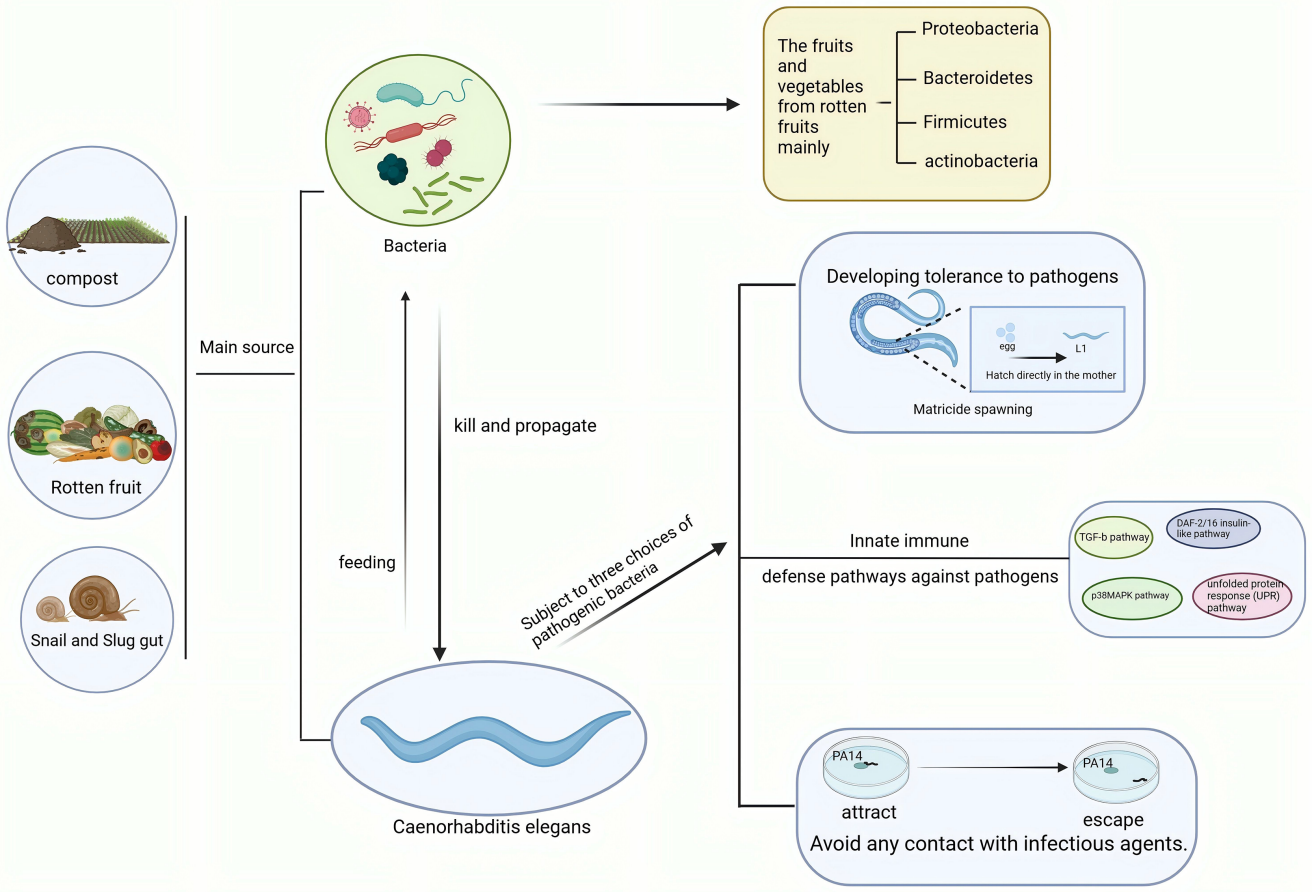
Interactions between *C. elegans* and pathogenic bacteria in ecological environments. *C. elegans* and pathogenic bacteria mainly originate from sources such as soil, compost, and the intestines of slugs. *C. elegans* feeds on bacteria, while pathogenic bacteria kill and spread through *C. elegans*. In response to pathogenic bacteria, *C. elegans* employs behaviors such as killing offspring, immune responses, and avoidance strategies. Created with BioRender.com.

The complex interplay between pathogenic bacteria and *C. elegans* elucidates a sophisticated network of coevolution and counter-behaviors that profoundly influence ecological dynamics. These interactions are further complicated by the element of predation. As bacterivorous organisms, *C. elegans* play a crucial role in shaping bacterial community dynamics within soil ecosystems (Fig. 2). Their predatory behavior can drive bacterial diversification, prompting bacterial populations to develop novel anti-predation strategies (Jiang et al., 2017). This dynamic interaction underscores the evolutionary pressures imposed by predation, potentially driving both *C. elegans* and bacteria to evolve adaptations that enhance their survival and reproductive success. Beyond direct predation, *C. elegans* can influence bacterial community structure through their selective feeding behaviors. Empirical studies have demonstrated that bacterial isolates exhibit differential susceptibility to predation by *C. elegans*, and such selective pressures can significantly influence the composition of rhizosphere microbial communities (Irshad & Yergeau, 2018). Consequently, this selective pressure may encourage the evolution of specific bacterial strains to develop traits that allow them to evade predation, thereby enhancing their ecological adaptability. These interactions demonstrate the complex feedback loop between *C. elegans* and bacteria, where the behavior of one significantly influences the evolutionary trajectory of the other.

*C. elegans* has evolved various mechanisms to respond to complex bacterial communities. Among these bacteria, some provide beneficial functions to *C. elegans*, while others pose threats to its growth and survival (Dirksen et al., 2016; Samuel et al., 2016). The dual role of the microbiome highlights that the ecological niche of *C. elegans* is not only determined by the physical environment but is also profoundly influenced by microbial interactions. Certain *Pseudomonas* and *Lactobacillus* strains enhance *C. elegans* resistance to pathogens, while antagonistic bacteria can limit its survival and growth, highlighting the impact of microbial diversity on *C. elegans* adaptability and ecological health (Dahan et al., 2020; Dirksen et al., 2016). To counter pathogen threats, *C. elegans* has evolved sensing and behavioral mechanisms, such as detecting quorum sensing signals from pathogens to influence developmental decisions like exiting the dauer stage (Werner et al., 2014). Its diverse chemoreceptors enable effective navigation and use of microbial resources, while chemical detoxification reduces microbial toxin effects, showcasing its adaptive strategies (Hartman et al., 2021; Lee et al., 2019).

Sensory and selective behaviors play a central role in these interactions. For instance, AWCON neurons recognize bacterial signals to adjust foraging behavior, helping *C. elegans* evade harmful bacteria and optimize dietary choices, which supports survival and influences microbial community dynamics (Sun et al., 2022). By selectively consuming non-pathogenic bacteria, *C. elegans* modulates microbiome composition, reducing pathogens and fostering a healthier ecosystem (Werner et al., 2014). Additionally, its foraging behavior promotes bacterial diversity, as different strains vary in susceptibility to predation, demonstrating how *C. elegans* adaptations shape both survival and broader ecosystem patterns (Ballestriero et al., 2016).

Pathogenic bacteria employ various strategies to influence *C. elegans* behavior, enhancing their survival and adaptability. For example, *Pseudomonas fluorescens* CHA0 produces secondary metabolites that reduce the nematode predation efficiency, giving the bacteria a competitive advantage in soil environments (Neidig et al., 2011). Pathogenic factors like phenazine and cyanide effectively kill *C. elegans*, thereby further enhancing bacterial survival and structuring microbial community dynamics (Chan et al., 2021; Jousset, 2012). This adaptive interplay, also often referred to as an “arms race,” reflects the continuous co-evolution of *C. elegans* and bacteria due to mutual pressures.

These bacterial defense mechanisms are not limited to natural environments and play an important role in agricultural settings as well. Within the realm of sustainable agriculture, bacterial biocontrol agents like *Bacillus* thuringiensis and *Bacillus* subtilis are employed to manage plant-parasitic *C. elegans*, serving as environmentally sustainable alternatives to chemical pesticides (Hu et al., 2020; Xia et al., 2011). Beyond their direct lethality to *C. elegans*, these bacteria are capable of inducing systemic resistance in plants, thereby indirectly influencing the interactions among plants, *C. elegans*, and bacteria (Kim et al., 2019). Probiotics, including Lacto *Bacillus* acidophilus, enhance the immunomodulation of *C. elegans*, thus providing resistance against pathogenic infections (Kim & Mylonakis, 2012). Such examples reveal the two sides of bacteria in ecosystems, being an essential nutritional source for *C. elegans* and a potentially deadly danger.

The interaction between microorganisms and *C. elegans* is not limited to antagonistic relationships but also includes mutualistic patterns. Some bacteria assist *C. elegans* in defending against threats posed by other pathogens. For example, *Pseudomonas* MYb11 can reduce viral transmission, while *Pseudomonas aeruginosa* PA14 decreases the susceptibility of *C. elegans* to the Orsay virus (Vassallo et al., 2024). This mutualistic interaction favored the survival of nematodes under complex conditions, proving again that cooperative and competitive strategies can coexist. Furthermore, some bacteria living in the gut of nematode *Acrobeloides maximus* may provide digesting or protective help against pathogen invasion to hosts, which further reinforces this ecological mutualism (Baquiran et al., 2013). Further, some pathogens infecting *C. elegans* may control the immune system and improve resistance to other pathogens, thus establishing a mutualistic relationship (Hajdú, Szathmári & Sőti, 2024). Studies have also shown that the interaction between pathogens and hosts can evolve into a mutualistic relationship through co-evolution. For example, the interaction between *C. elegans* and the mildly parasitic bacterium *Enterococcus faecalis* demonstrates that, with bacterial evolution, they can provide protection against pathogens like *Staphylococcus aureus*, and this protective effect, in turn, promotes host adaptation to the bacteria, establishing a mutualistic relationship (Rafaluk-Mohr et al., 2018). This phenomenon indicates the interaction between host and pathogen is not fixed but changed under certain environmental conditions and selective pressure. In plant-microbe interaction, the coexistence between pathogens and mutualistic microbes also shows a complex dynamic. When plants are under attack by pathogens, they may increase their defense mechanisms through interactions with mutualistic microbes, which affects plant growth and survival, as well as the pathogenicity and transmission of pathogens (Marchetto & Power, 2018). Therefore, this complex interaction is very important to reveal the dynamic balance of ecosystems and biodiversity.

## Conclusions

*C. elegans* serves as a valuable model for studying host-pathogen interactions, offering key insights into immune responses, behavioral adaptations, and co-evolutionary dynamics with pathogenic bacteria. It employs a range of survival strategies, including altered foraging behaviors, pathogen avoidance, and learned behavioral responses that can be transmitted across generations. In addition to these behavioral defenses, *C. elegans* possesses an innate immune system that helps detect and neutralize microbial threats. However, the role of symbiotic bacteria in immune defense and microbial community regulation remains less understood and requires further investigation.

Future research should explore how *C. elegans* reshapes microbial populations, particularly the interactions between pathogenic and non-pathogenic bacteria. Investigating the feedback loops between *C. elegans* behavior, microbial diversity, and immune responses could deepen our understanding of its ecological impact. Expanding research in these areas will not only enhance our knowledge of host-microbe interactions but also provide broader implications for human health, disease resistance, and environmental management.

## Additional Information and Declarations

### Data Availability

The following information was supplied regarding data availability:

This is a literature review.
